# Integrative multi-omics analyses identify PKD1 and SLC2A4 as genetically supported glycolysis-related candidate genes for rheumatoid arthritis

**DOI:** 10.3389/fimmu.2025.1691663

**Published:** 2026-01-22

**Authors:** Xinyu A, Pengfei Xin, Lin Zheng, Bo Xu, Jianye Wang, Songtao Sun, Jun Xie, Chenxin Gao, Peijun Pan, Guowei Qiu, Lang Jin, Jun Shen, Xirui Xu, Yiwei Cheng, Shaoqiang Pei, Lei Ran, Yanqin Bian, Lianbo Xiao

**Affiliations:** 1 Shanghai University of Traditional Chinese Medicine, Shanghai, China; 2 The Research Institute for Joint Diseases, Shanghai Academy of Traditional Chinese Medicine, Shanghai, China; 3 Jiangxi University of Traditional Chinese Medicine Affiliated Hospital, Nanchang, China; 4 Shanghai Guanghua Hospital of Integrative Medicine, Shanghai, China

**Keywords:** DNA methylation, gene expression, glycolysis, Mendelian randomization, multi-omics, PKD1, rheumatoid arthritis, SLC2A4

## Abstract

**Introduction:**

Glycolytic reprogramming has been implicated in rheumatoid arthritis (RA) pathogenesis, yet the underlying causal genes and epigenetic mechanisms remain unclear. This study aimed to systematically identify glycolysis-related genes and their methylation-regulated expression that may causally influence RA susceptibility.

**Methods:**

We conducted a multi-omics Mendelian randomization (MR) analysis integrating genome-wide association study (GWAS) summary statistics for RA (FinnGen, UK Biobank, GCST90129453) with quantitative trait loci (QTLs) for blood-derived methylation (mQTL), expression (eQTL), and protein abundance (pQTL). Summary-data-based Mendelian randomization (SMR) and colocalization analyses were used to identify causal molecular signatures linking DNA methylation, gene expression, and protein abundance with RA risk. Replication was performed in independent RA cohorts. In addition, qPCR validation was conducted in an independent whole-blood cohort (30 RA patients and 30 healthy controls).

**Results:**

SMR identified 129 CpG sites (75 genes), 28 transcripts, and 9 proteins significantly associated with RA risk. Seven glycolytic genes—PKD1, SLC2A4, ALAS1, ALDH7A1, LRFN3, PFKFB2, and PYGB—showed consistent evidence across methylation, expression, and GWAS datasets. Notably, hypomethylation at cg07036112 (PKD1; OR = 0.68, 95% CI: 0.59–0.78) and cg06891043 (SLC2A4; OR = 0.92, 95% CI: 0.89–0.96) was associated with increased gene expression and increased RA susceptibility. Colocalization supported shared causal variants at these loci (PP.H4 > 0.5). Additional signals included cg13241645 (ALAS1; OR = 0.72, 95% CI: 0.65–0.80) and cg01380361 (PFKFB2; OR = 1.33, 95% CI: 1.17–1.51). qPCR confirmed increased PKD1 and SLC2A4 mRNA expression in RA compared with healthy controls.

**Discussion:**

This integrative multi-omics MR framework supports an epigenetically mediated contribution of glycolysis-related regulation to RA susceptibility and nominates PKD1 and SLC2A4 as robust genetically supported candidate genes. These findings highlight methylation-linked transcriptional changes in glycolysis-related pathways implicated in RA and suggest potential biomarkers and therapeutic targets.

## Introduction

Rheumatoid arthritis (RA) is a chronic autoimmune disorder marked by persistent synovitis, progressive joint damage, and systemic inflammation, affecting approximately 0.5–1% of the global population (1). Despite advances in biologics and targeted therapies, many patients continue to experience disease flares and irreversible joint destruction. Beyond immune dysregulation, metabolic remodeling has emerged as a fundamental component of RA pathogenesis. Synovial fibroblasts and infiltrating immune cells reprogram their metabolism in response to inflammatory and hypoxic microenvironments, switching from oxidative phosphorylation to aerobic glycolysis (2, 3). This glycolytic shift supports energy-intensive processes like proliferation, matrix degradation, and cytokine production, which exacerbate joint inflammation. Metabolites such as lactate and succinate also accumulate locally, serving as signaling molecules that amplify inflammation and matrix remodeling (4). Consequently, metabolic adaptation not only sustains pathogenic cell function but also reshapes the RA synovial ecosystem.

Fibroblast-like synoviocytes (FLS) in RA exhibit a highly activated phenotype that mimics tumor-like behavior, including hyperproliferation, invasiveness, and resistance to apoptosis (5). This phenotype is driven by increased glucose uptake and elevated expression of glycolytic enzymes, such as HK2, LDHA, and PFKFB3 (6). Similarly, CD4+ and CD8+ T cells within the inflamed synovium undergo metabolic rewiring that enhances their effector functions, partly through HIF-1α and mTORC1-mediated pathways (7, 8). Various regulators—including metabolic enzymes and signaling proteins—can influence glycolytic switching and proinflammatory phenotypes in immune cells (7). Glycolytic inhibition in both FLS and immune cells has been shown to reduce inflammatory mediator secretion and cell migration, supporting the rationale for exploring glycolysis-targeted strategies in RA (9, 10). However, the upstream regulatory architecture linking genetic susceptibility to these glycolytic phenotypes in RA remains incompletely understood.

Recent advances in multi-omics integration have enabled systematic exploration of gene regulation in complex diseases. Summary-data-based Mendelian randomization (SMR) leverages genetic variants as instruments to infer causal links between molecular traits (e.g., DNA methylation, gene expression, protein abundance) and disease outcomes (11). SMR approaches that integrate QTL datasets—including methylation quantitative trait loci (mQTLs), expression quantitative trait loci (eQTLs), and protein quantitative trait loci (pQTLs)—with GWAS summary statistics can reveal molecular features causally associated with RA. While this strategy has been successfully applied to investigate autophagy and immune pathways in RA, its application to glycolysis remains limited. In this study, we employed a multi-layer SMR framework to investigate whether glycolysis-related molecular features are causally linked to RA risk. By integrating GWAS data from the FinnGen, UK Biobank, and GCST90129453 cohorts with multi-omics QTL datasets, we aimed to identify key methylation sites, transcripts, and proteins involved in glycolysis that may act as upstream drivers of RA pathogenesis. Here, we employ a multi-omics MR framework to systematically investigate causal roles of epigenetically regulated glycolytic genes in RA.

## Materials and methods

### Data sources

**Figure 1:**
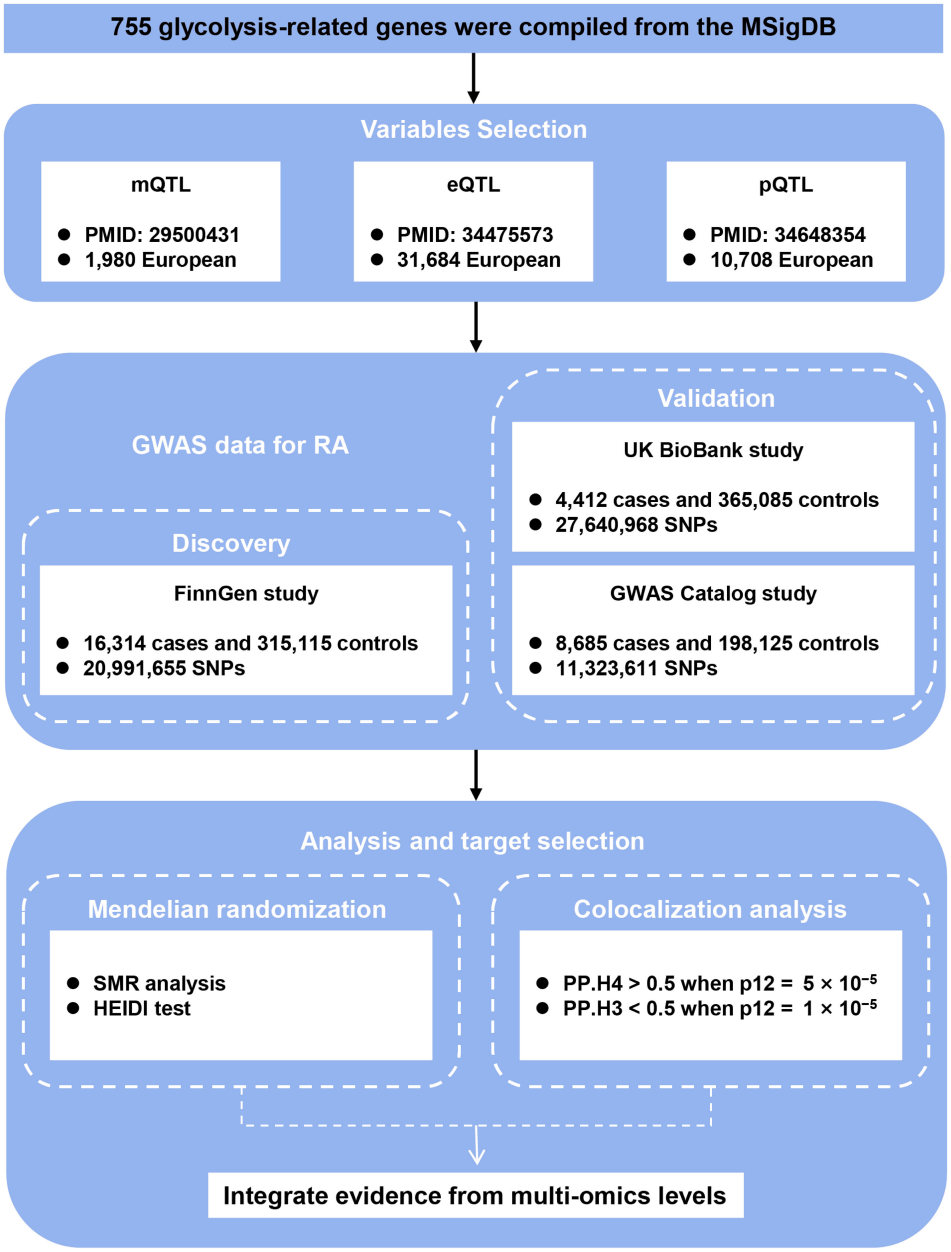
Study design. Schematic overview of integrating GWAS, mQTL, eQTL, and pQTL data to identify causal glycolysis-related genes in RA. QTL, quantitative trait loci; RA, rheumatoid arthritis; SNPs, single nucleotide polymorphisms; SMR, summary-data-based Mendelian randomization; HEIDI, heterogeneity in dependent instrument; PP.H3, posterior probability of H3; PP.H4, posterior probability of H4.

The complete study design, including data sources and multi-omics integration strategy, is summarized in **Figure 1**. A total of 755 unique glycolysis-related genes were compiled from 22 gene sets retrieved from the Molecular Signatures Database (MSigDB; https://www.gsea-msigdb.org/) using the keyword “glycolysis” (12), after merging and removing duplicates.

Three independent RA genome-wide association study (GWAS) datasets were used. The primary discovery cohort was obtained from the FinnGen consortium (Release R12, GWAS ID: M13_RHEUMA), including 16,314 RA cases and 315,115 controls of European ancestry. For validation, we used two independent RA cohorts: (1) the UK Biobank dataset from PheWeb (GWAS ID: PheCode 714.1 (4,412 cases and 365,085 controls) and (2) the GWAS Catalog dataset GCST90129453 (8,685 cases and 198,125 controls) (**Table 1**). All datasets were publicly available and based on individuals of European descent.

**Table 1:** GWAS cohort information.

Trait	Disease	GWAS ID	Sample size (case/control)	SNP Count
RA	Rheumatoid Arthritis	M13_RHEUMA	16314/315115	20,991,655
RA	Rheumatoid Arthritis	PheCode 714.1	4412/365085	27,640,968
RA	Rheumatoid Arthritis	GCST90129453	8685/198125	11,323,611

We obtained blood-based cis-eQTL summary statistics from the eQTLGen Consortium, encompassing data from 31,684 individuals (13), derived blood-based mQTL data from a meta-analysis of two European cohorts—the Brisbane Systems Genetics Study (n = 614) and the Lothian Birth Cohorts (n = 1,366) (14) that were standardized using the minfi package with BMIQ normalization, and extracted blood-based pQTL summary statistics from a large-scale proteogenomic study involving 10,708 European participants, as reported by Pietzner et al (15).

### SMR analysis

We conducted SMR analysis using the SMR software (v1.3.1), a method that leverages top associated cis-QTLs to evaluate putative causal relationships between molecular features—methylation (mQTL), gene expression (eQTL), protein abundance (pQTL)—and RA risk. SMR is particularly suited for integrating data from large, non-overlapping QTL and GWAS datasets.

For each QTL dataset, cis-variants within ±1000 kb of each probe were selected as instruments, with a genome-wide significance threshold (p < 5.0 × 10^−8^). SNPs with allele frequency differences exceeding 0.2 between QTL and GWAS datasets were excluded; up to 5% mismatch across all SNPs was tolerated for inclusion. SMR analysis was performed independently for mQTL-GWAS, eQTL-GWAS, and pQTL-GWAS combinations. To account for potential linkage and pleiotropy, the HEIDI (Heterogeneity In Dependent Instruments) test was applied, with p_HEIDI > 0.05 indicating no heterogeneity and supporting a causal interpretation.

We also implemented a multi-SNP version of the SMR test (--smr-multi), which considers all SNPs in the QTL probe window (default window =500 kb) with p < 5.0 × 10^−8^and LD r² < 0.9. Final candidate loci were defined by the joint significance of p_SMR_multi < 0.05 and p_HEIDI > 0.05. To validate positive findings, replication SMR analyses were performed using two independent RA GWAS datasets: the UK Biobank (PheWeb 714.1) and GCST90129453, using the same significance and heterogeneity thresholds.

In addition to evaluating QTL-disease associations, we used SMR to infer causal relationships between methylation and gene expression (mQTL–eQTL), as well as between gene expression and protein abundance (eQTL–pQTL). For mQTL–eQTL SMR, CpG sites were treated as exposures and gene transcripts as outcomes, providing insights into epigenetic regulation of expression. For eQTL–pQTL SMR, transcript levels were modeled as exposures and protein abundance as outcomes. These integrative analyses aimed to uncover regulatory cascades and prioritize key glycolytic genes contributing to RA susceptibility.

### Co-localization analysis

To verify whether QTL signals and RA GWAS associations share a common causal variant, we performed co-localization analysis using the Bayesian framework implemented in the R package coloc. This method estimates the posterior probability for five mutually exclusive hypotheses: H0 (no association with either trait), H1 (association with QTL only), H2 (association with GWAS only), H3 (both traits associated but with distinct causal variants), and H4 (both traits associated and sharing the same causal variant). Evidence for co-localization was defined by either PP.H4 > 0.5 when the prior probability p12 was set to 5 × 10^−5^, or PP.H3 < 0.5 when p12 was set to 1 × 10^−5^ (16).

Co-localization analyses were independently conducted for mQTL-GWAS, eQTL-GWAS, and pQTL-GWAS pairs. Following published protocols, genomic regions were using the same ±500 kb (mQTL) or ±1000 kb (eQTL/pQTL) windows described above (17–19). Summary-level data for each QTL-GWAS pair were extracted and formatted according to coloc requirements. Only loci with significant SMR results and without evidence of heterogeneity (p_HEIDI > 0.05) were included in the co-localization step to reduce spurious associations.

### Ethics approval and consent

Ethics review statement. This study involving human participants was reviewed and approved by the Ethics Committee of Shanghai Guanghua Hospital of Integrative Medicine. All procedures complied with the Declaration of Helsinki and relevant national regulations. All participants (or legal guardians, where applicable) provided written informed consent prior to enrollment and prior to collection of study specimens (peripheral blood samples for whole-blood RNA extraction). Recruitment, sample collection, and clinical assessments were conducted at Shanghai Guanghua Hospital of Integrative Medicine.

### Patient cohorts and whole-blood qPCR validation

We recruited 30 RA patients and 30 age- and sex-matched healthy controls from Shanghai Guanghua Hospital of Integrative Medicine. Peripheral blood was collected into EDTA tubes. Total RNA was extracted from whole blood, and 1 μg RNA was reverse transcribed into cDNA. PKD1 and SLC2A4 mRNA levels were quantified by SYBR Green–based qPCR using ACTB (β-actin) as the reference gene. Relative expression was calculated by the 2^–ΔCt method (ΔCt = Ct(target) – Ct(ACTB)).

### Statistical analysis

We conducted statistical analyses in R (v4.4.3) for all computational/omics components and in GraphPad Prism (v10.4.1) for qPCR validation and graphing. We generated Manhattan plots with “ggplot2” and “ggrepel”, and forest plots with “forestplot”. We produced locus and effect plots using modified SMRLocusPlot and SMREffectPlot functions from Zhu et al (20). For the whole-blood qPCR dataset, relative expression values (2^–ΔCt) of PKD1 and SLC2A4 were first assessed for normality using the Shapiro–Wilk test. Group comparisons between RA and HC were performed using two-tailed unpaired t-tests when normality was not grossly violated; otherwise, the Mann–Whitney U test was applied. qPCR data are summarized as mean ± standard deviation (SD). Unless stated otherwise, P values are two-sided with α = 0.05.

## Results

### Association of glycolysis-related CpG methylation with RA risk

To investigate the potential association between CpG site methylation and RA, we performed SMR analysis by integrating whole-blood mQTL data with GWAS summary statistics from the FinnGen R12 cohort. A total of 129 CpG sites located within 75 glycolysis-related genes were identified as significantly associated with RA (p_SMR < 0.05, p_SMR_multi < 0.05, p_HEIDI > 0.05), as shown in **Supplementary Table S1**.

**Figure 2:**
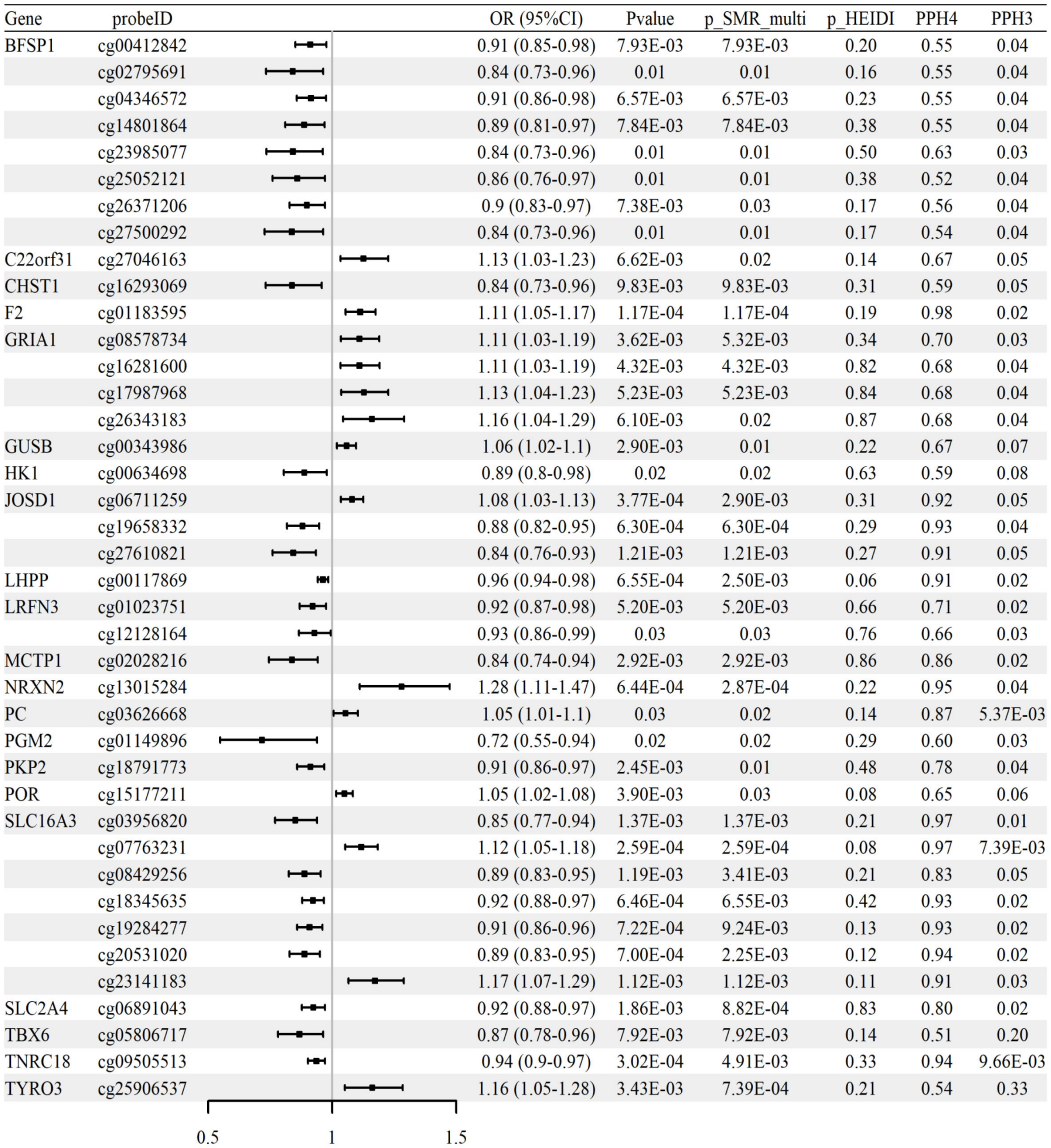
Associations between methylation of glycolysis-related CpG sites and RA risk (whole-blood mQTL meta-analysis; FinnGen R12 RA GWAS). Forest plot of 40 CpG sites with strong colocalization evidence (PP.H4 > 0.5, PP.H3 < 0.5); ORs and 95% CIs are shown.

Colocalization analysis further revealed that 40 of these CpG sites, corresponding to 21 genes, showed strong evidence of sharing a causal variant with RA GWAS signals (PP.H4 > 0.5 and PP.H3 < 0.5). These colocalized signals are summarized comprehensively in **Figure 2**, with detailed representative examples illustrated in **Supplementary Figure S1**. Among the notable findings, methylation at cg06711259 (located in JOSD1) was positively associated with RA risk (OR = 1.08, 95% CI [1.03–1.13]), while another CpG within the same gene, cg19658332, exhibited a negative association (OR = 0.88, 95% CI [0.82–0.95]).

In replication analyses using GWAS datasets from the UK Biobank and GCST90129453 cohorts, we did not observe significant associations meeting validation criteria in the UK Biobank dataset. However, we validated six CpG sites—cg26105232 (IL2RA), cg12444328 (LHX9), cg20945261 (NUP210), cg07568117 and cg07036112 (PKD1), and cg23514324 (PPARG)—in the GCST90129453 cohort (p_SMR < 0.05, p_SMR_multi < 0.05, and p_HEIDI > 0.05). These replicated associations are summarized in **Table 2**, with full results available in **Supplementary Tables S2–S3**. Collectively, these methylation changes suggest epigenetic modulation of glycolytic pathways, potentially exacerbating RA inflammation.

**Table 2:** SMR validation of glycolysis-related mQTL and eQTL signals for rheumatoid arthritis in independent RA GWAS cohorts.

QTL type¹	Probe ID/Gene ID	Symbol	p_SMR	p_SMR_multi	p_HEIDI	OR_SMR (95% CI)
mQTL^#^	cg26105232	IL2RA	4.41E-02	4.41E-02	7.25E-01	0.84 (0.71–1.00)
mQTL^#^	cg12444328	LHX9	3.94E-02	4.73E-02	7.30E-01	0.87 (0.77–0.99)
mQTL^#^	cg20945261	NUP210	4.11E-02	1.45E-02	6.53E-01	1.10 (1.00–1.21)
mQTL^#^	cg07568117	PKD1	2.48E-02	3.76E-02	7.19E-01	0.91 (0.85–0.99)
mQTL^#^	cg07036112	PKD1	4.65E-02	4.65E-02	8.87E-01	0.87 (0.76–1.00)
mQTL^#^	cg23514324	PPARG	3.53E-03	8.82E-03	7.42E-01	0.91 (0.86–0.97)
eQTL^$^	ENSG00000162976	SLC66A3	1.27E-03	3.30E-02	9.02E-01	1.26 (1.09–1.44)
eQTL^#^	ENSG00000008710	PKD1	3.39E-02	3.39E-02	6.48E-01	1.39 (1.03–1.87)
eQTL^#^	ENSG00000162976	SLC66A3	4.67E-04	5.75E-03	2.71E-01	1.20 (1.08–1.33)

^1^: Superscripts denote the RA GWAS cohort used in the SMR validation: ^#^: GCST90129453 RA cohort; ^$^: UK Biobank RA cohort;

### Association of glycolysis-related gene expression with RA risk

**Figure 3:**
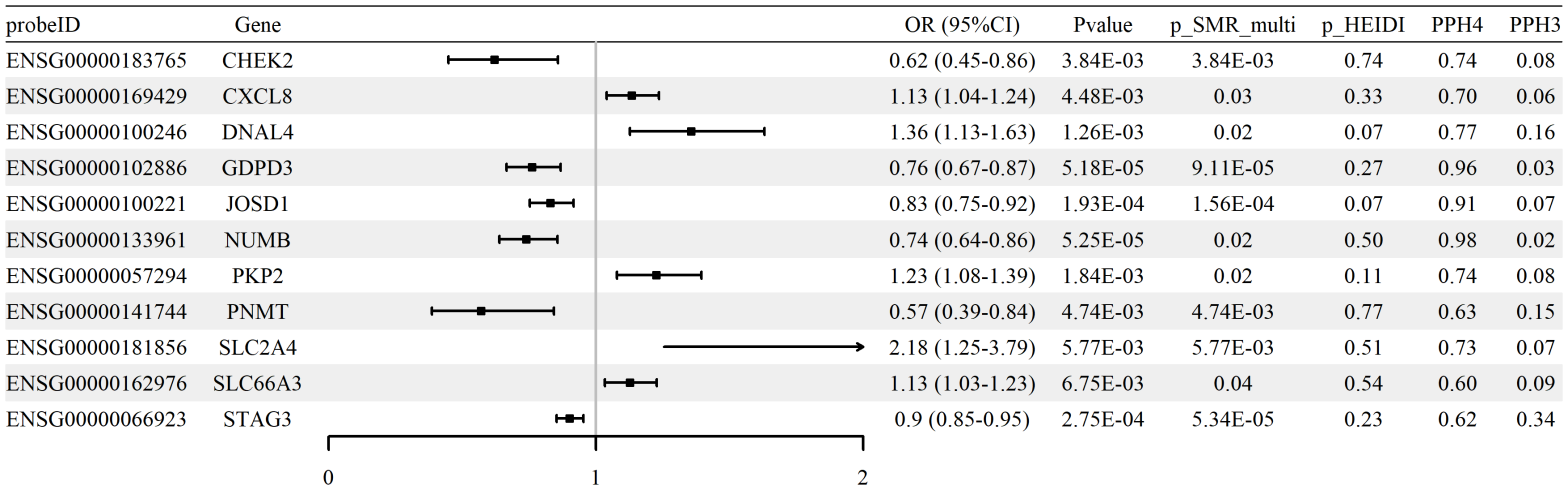
Associations between expression of glycolysis-related genes and RA risk (blood cis-eQTLGen; FinnGen R12 RA GWAS). Forest plot of 11 genes meeting SMR/HEIDI criteria; ORs and 95% CIs are shown, with colocalization support indicated (PP.H4 > 0.5).

We performed SMR analysis to examine the association between glycolysis-related gene expression (eQTLs) and RA using the FinnGen R12 GWAS dataset. A total of 28 genes met the criteria for statistical significance (p_SMR < 0.05, p_SMR_multi < 0.05, and p_HEIDI > 0.05), with detailed results provided in **Supplementary Table S4**. Among these, 11 genes showed evidence of colocalization between gene expression and RA GWAS signals, defined by posterior probabilities PP.H4 > 0.5 and PP.H3 < 0.5. These genes and their corresponding SMR estimates are summarized in **Supplementary Table S5**, and the associations are visualized in **Figure 3**. Colocalization examples are shown in **Supplementary Figure S2**. Together, these expression patterns are consistent with glycolytic reprogramming in RA and implicate transport and signaling nodes alongside counter-regulatory effects.

Effect direction was evaluated using odds ratios (ORs). Increased expression of genes such as SLC2A4 (OR = 2.18, 95% CI [1.25–3.79]) and CXCL8 (OR = 1.13, 95% CI [1.04–1.24]) was associated with higher RA risk, whereas higher expression of CHEK2 (OR = 0.62, 95% CI [0.45–0.86]) and PNMT (OR = 0.57, 95% CI [0.39–0.84]) was associated with lower risk.

Validation analyses were performed using two independent GWAS datasets (UK Biobank and GCST90129453). Among the 28 identified genes, significant associations for SLC66A3 were consistently observed across both datasets, and PKD1 was also validated in the GCST90129453 cohort. Replication analysis results are summarized in **Table 2**; comprehensive statistical details are available in **Supplementary Tables S6, S7**.

### Association of glycolysis-related protein abundance with RA risk

To evaluate whether protein abundance of glycolysis-related genes is associated with RA, SMR analysis was conducted using blood-derived pQTL data and FinnGen R12 GWAS summary statistics. A total of nine proteins, including AGAP2, B3GALT6, FBP1, INSL5, MDK, PGP, SIRPB1, TGFBI, and TYRO3, were identified as significantly associated with RA (p_SMR < 0.05, p_SMR_multi < 0.05, and p_HEIDI > 0.05). Full results are reported in **Supplementary Table S8**. Among them, five proteins (TGFBI, SIRPB1, FBP1, TYRO3, and MDK) showed positive associations with RA risk, while four (AGAP2, B3GALT6, INSL5, and PGP) were negatively associated.

**Figure 4:**
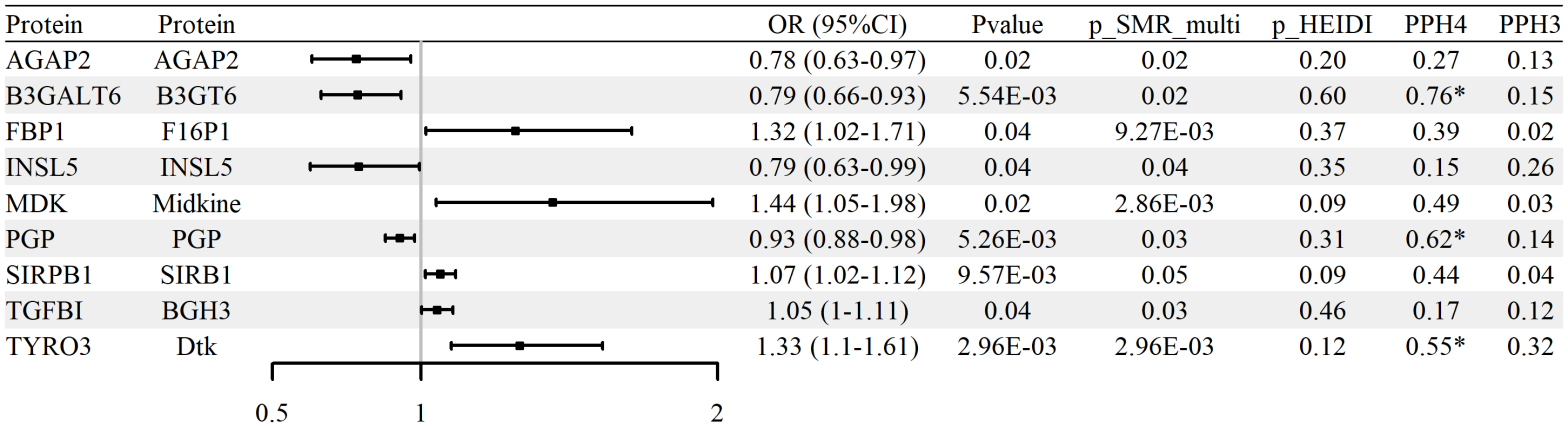
Associations between glycolysis-related protein abundance and RA risk (blood pQTL; FinnGen R12 RA GWAS). Forest plot of nine proteins identified by pQTL–GWAS SMR; ORs and 95% CIs are shown.

Colocalization analysis indicated that three proteins (B3GALT6, TYRO3, and PGP) exhibited strong evidence of shared causal variants with RA GWAS loci (PP.H4 > 0.5 and PP.H3 < 0.5). These results are summarized in **Supplementary Table S9** and illustrated in **Figure 4** and **Supplementary Figure S3**. No statistically significant associations were confirmed upon replication in either the UK Biobank or GCST90129453 RA cohorts (p_SMR ≥ 0.05 or p_HEIDI ≤ 0.05), with replication results detailed in **Supplementary Tables S10–S11**. In aggregate, the protein-level signals nominate biologically plausible candidates but warrant cautious interpretation and validation across independent cohorts.

### Integration of blood mQTL and eQTL data related to glycolysis and RA GWAS

To assess whether CpG site methylation may regulate the expression of glycolysis-related genes implicated in RA, SMR analysis was performed using blood mQTLs as exposures and eQTLs as outcomes. The analysis focused on genes previously identified through independent mQTL–GWAS and eQTL–GWAS associations. Significant associations between methylation and expression were identified for seven genes—PKD1, SLC2A4, ALAS1, ALDH7A1, LRFN3, PFKFB2, and PYGB—covering 12 distinct CpG sites. SMR associations met the criteria of p_SMR < 0.05, p_SMR_multi < 0.05, and p_HEIDI > 0.05. The complete analysis results are presented in **Supplementary Table S12**, and selected findings are shown in **Supplementary Table S5**.

Representative examples include cg07036112 at the PKD1 locus (OR = 0.68, 95% CI 0.59–0.78), where lower methylation was linked to increased PKD1 expression and subsequently increased RA risk. Similarly, cg06891043 at the SLC2A4 locus (OR = 0.92, 95% CI 0.89–0.96) demonstrated hypomethylation associated with higher SLC2A4 expression and increased RA risk. Additional signals—cg13241645 at ALAS1 (OR = 0.72, 95% CI 0.65–0.80), cg01380361 at PFKFB2 (OR = 1.33, 95% CI 1.17–1.51), and multiple CpGs in PYGB—consistently indicated epigenetic mechanisms driving glycolytic gene expression that collectively contribute to RA susceptibility (**Table 3**).

**Table 3:** mQTL - eQTL SMR analysis results: potential regulatory relationships.

Expo ID	Outco Gene	p_SMR	p_SMR_multi	p HEIDI	OR SMR (95% CI)
cg13241645	ALAS1	3.39E-09	3.39E-09	8.15E-01	0.72(0.65-0.8)
cg22547559	ALDH7A1	9.51E-15	9.51E-15	5.98E-01	0.35(0.27-0.46)
cg15658249	LRFN3	6.74E-08	6.74E-08	4.06E-01	2.9(1.97-4.26)
cg01380361	PFKFB2	8.85E-06	8.85E-06	2.24E-01	1.33(1.17-1.51)
cg07036112	PKD1	2.24E-08	2.24E-08	7.43E-02	0.68(0.59-0.78)
cg02174639	PYGB	1.51E-10	1.51E-10	6.98E-01	0.03(0.01-0.08)
cg02738255	PYGB	5.51E-11	5.51E-11	1.43E-01	0.03(0.01-0.09)
cg04267284	PYGB	7.57E-10	7.57E-10	8.67E-01	0.02(0.01-0.08)
cg04348305	PYGB	5.35E-09	5.35E-09	4.10E-01	0.03(0.01-0.09)
cg06421707	PYGB	2.87E-08	2.87E-08	6.92E-01	65.29(14.92-285.66)
cg07328115	PYGB	2.91E-17	2.91E-17	3.73E-01	0.07(0.04-0.13)
cg06891043	SLC2A4	1.50E-05	4.11E-04	7.54E-02	0.92(0.89-0.96)

### Integration of RA GWAS with glycolysis-related pQTL and eQTL data

**Figure 5:**
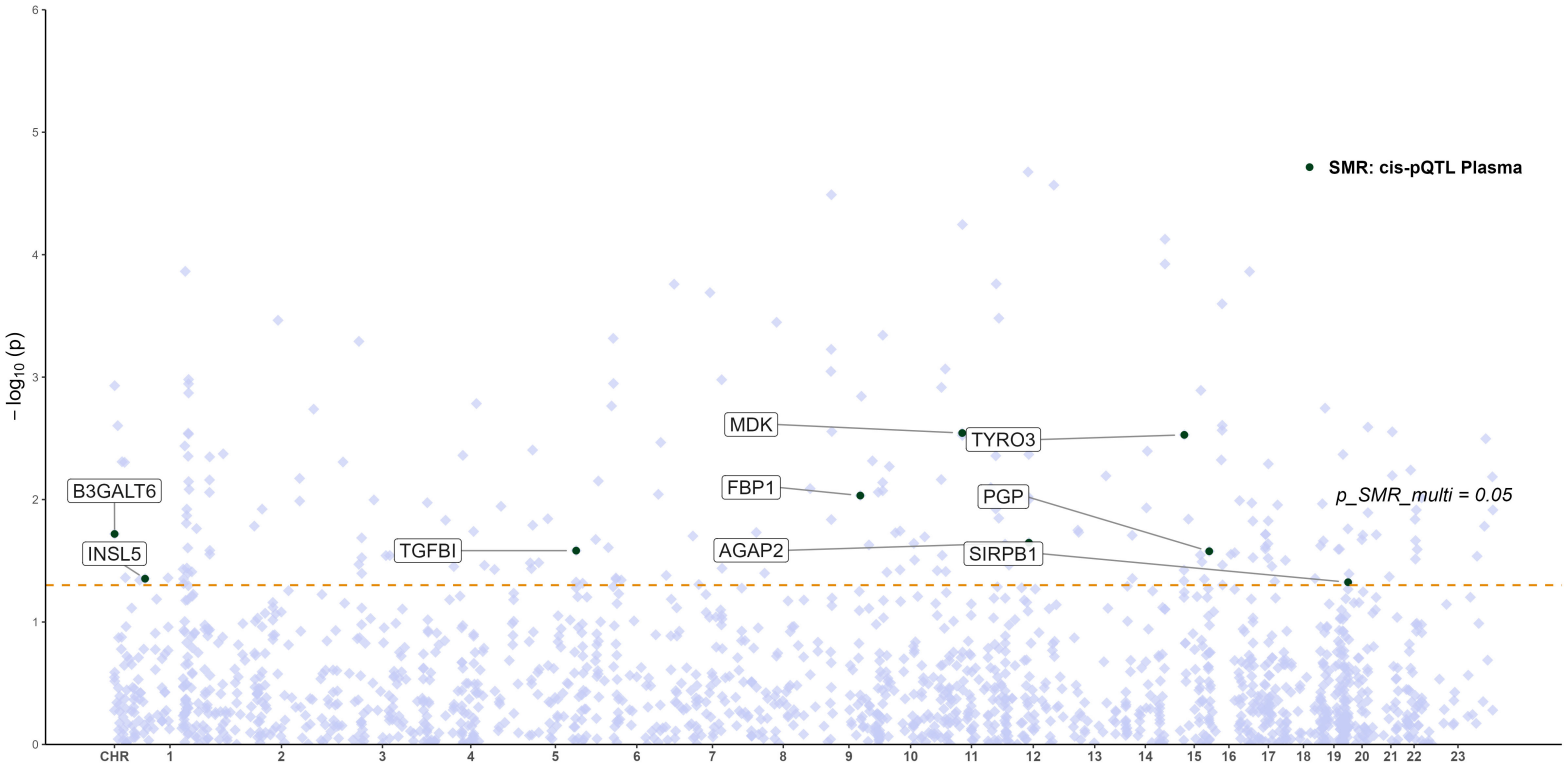
Manhattan plot of pQTL–GWAS SMR results for RA (blood pQTL; FinnGen R12 RA GWAS). Proteins meeting the SMR multi-test threshold are labeled.

To explore whether the expression of key glycolysis-related genes affects protein abundance relevant to RA, we performed an integrative SMR analysis combining pQTL and eQTL data. However, no statistically significant causal relationships (p_SMR < 0.05, p_SMR_multi < 0.05, and p_HEIDI > 0.05) were identified between gene expression levels and downstream protein abundance. Therefore, no eQTL–pQTL SMR analysis was pursued further. The distribution of pQTL–RA associations across chromosomes is displayed in **Figure 5**, highlighting the genomic locations of key proteins (e.g., TYRO3, PGP, MDK) that reached nominal significance (p_SMR_multi < 0.05), although not validated at the multi-omics integration level.

### Multi-omics integration and chromosomal distribution of candidate glycolytic genes associated with RA

**Figure 6:**
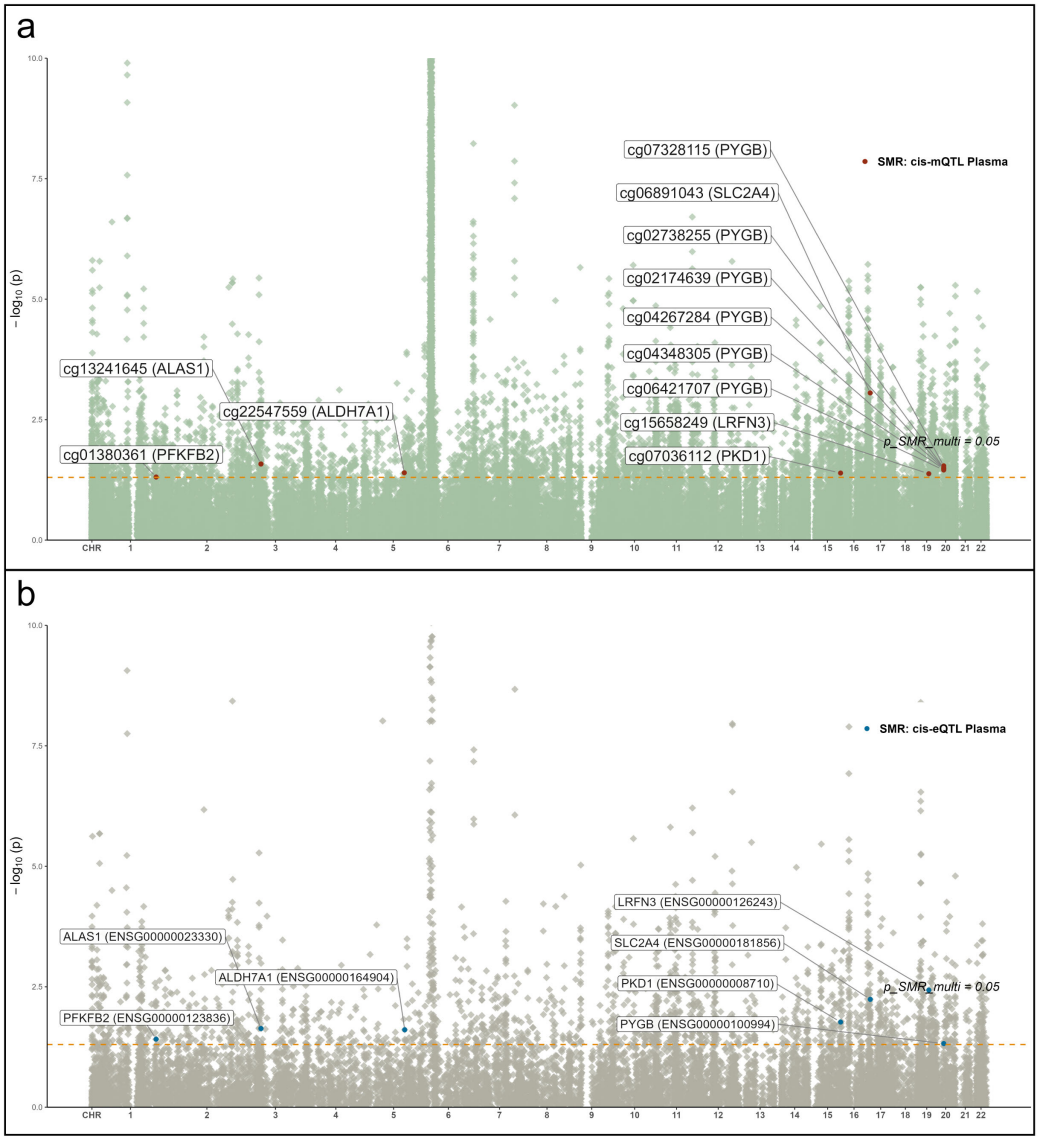
Manhattan plots of QTL–GWAS SMR results for RA (FinnGen R12 RA GWAS). **(a)** whole-blood mQTL; **(b)** blood cis-eQTL. Key loci (e.g., PKD1 and SLC2A4) are labeled.

To identify glycolysis-related candidate genes supported by convergent evidence across molecular layers, we integrated summary data from mQTL, eQTL, and RA GWAS using SMR analysis (**Figures 6a, b**). Seven genes—*ALAS1, ALDH7A1, LRFN3, PFKFB2, PKD1, PYGB*, and *SLC2A4*—demonstrated significant associations across at least two omics layers and mapped to distinct genomic loci. These results revealed directionally consistent associations between mQTL and eQTL signals, with partial protein-level trends reinforcing biological plausibility. Notably, cg07036112 (*PKD1*) and cg06891043 (*SLC2A4*) exhibited suggestive colocalization evidence with RA signals (PP.H4 > 0.5 and PP.H3 < 0.5), indicating potential shared causal variants. For instance, lower methylation at cg07036112 was associated with elevated PKD1 expression, consequently increasing RA risk (OR = 0.68, 95% CI 0.59–0.78), suggesting an epigenetically mediated activation mechanism. Similarly, for *ALAS1* and *ALDH7A1*, hypomethylation correlated with upregulated gene expression, with ALAS1 (OR = 0.72, 95% CI 0.65–0.80) and ALDH7A1 (OR = 0.35, 95% CI 0.27–0.46) showing significant associations with RA.

### Colocalization and SMR effect size analyses of PKD1 and SLC2A4

**Figure 7:**
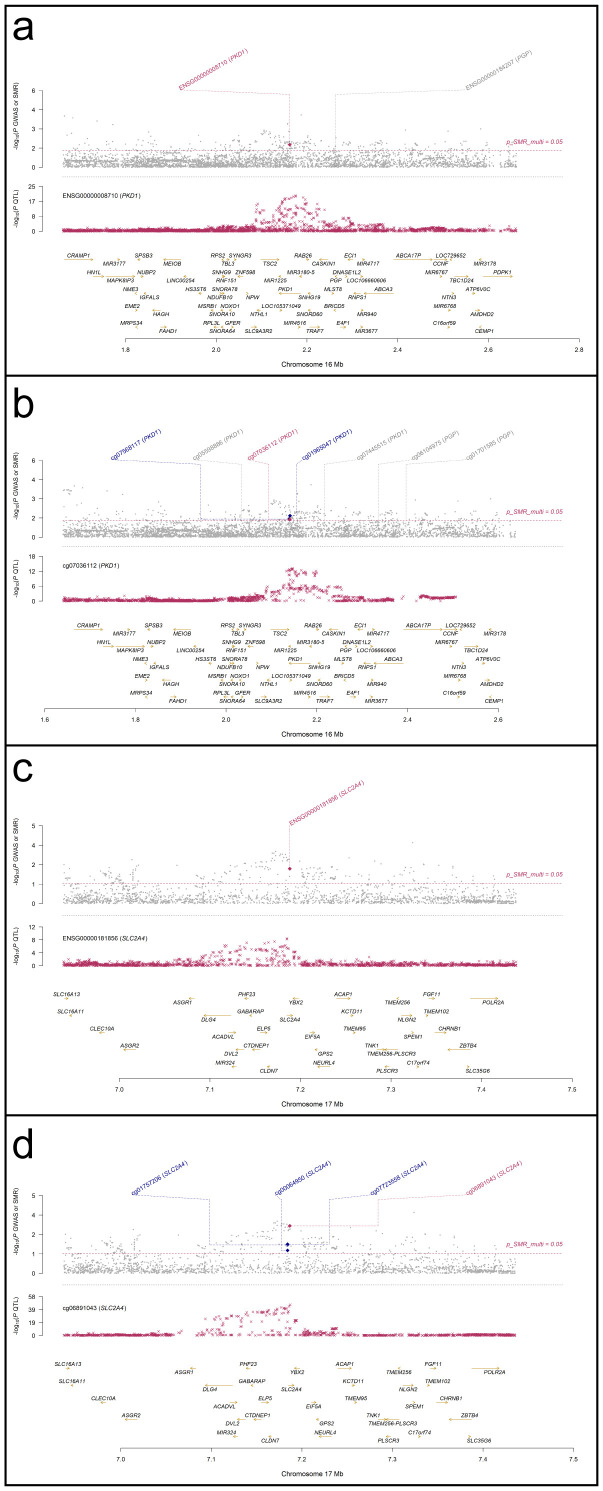
Regional association and colocalization plots for PKD1 and SLC2A4 (blood mQTL/eQTL and FinnGen R12 RA GWAS). **(a, b)** PKD1; **(c, d)** SLC2A4. Evidence of shared causal variants is indicated by PP.H4 > 0.5.

**Figure 8:**
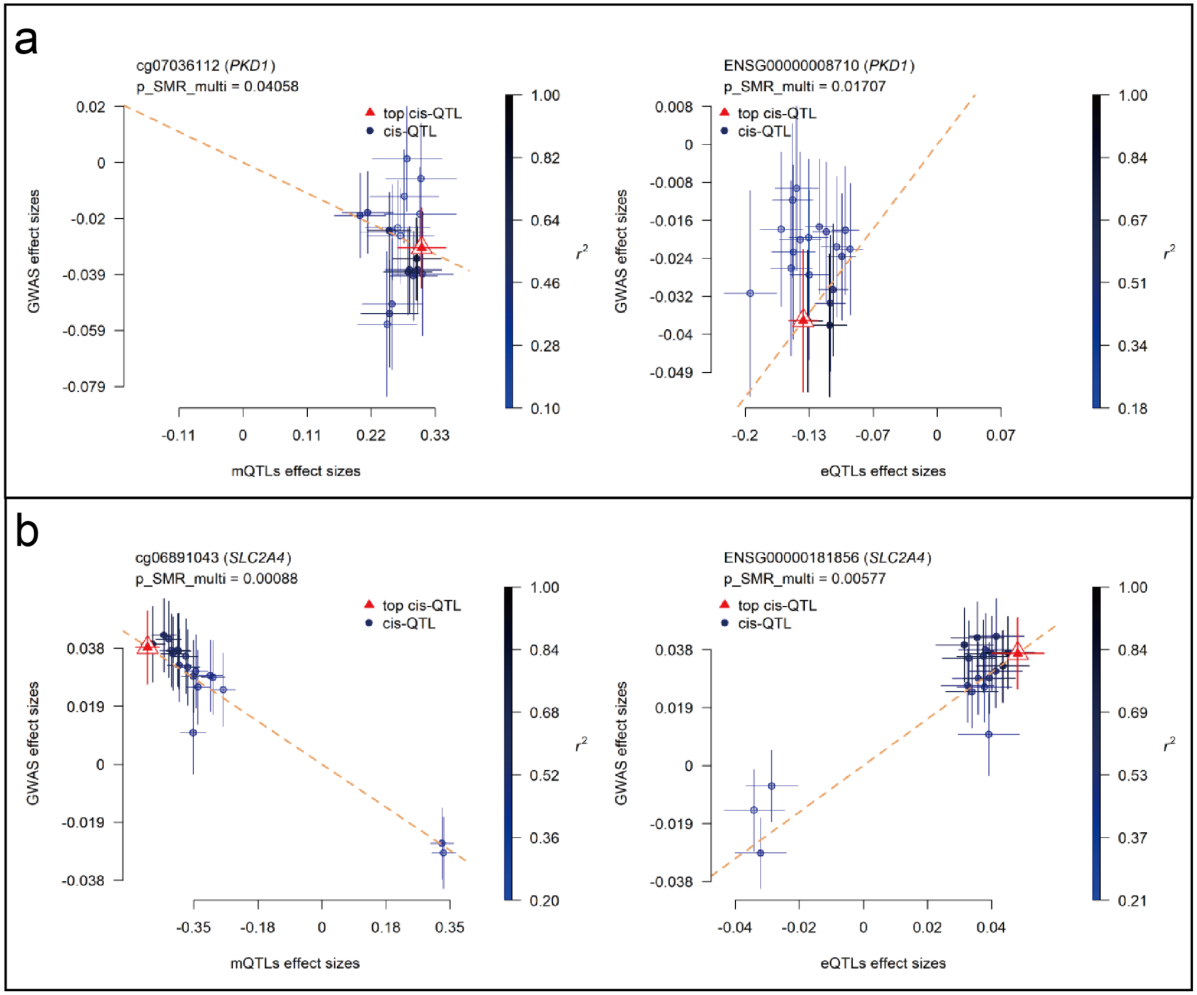
SMR EffectPlot analysis for PKD1 and SLC2A4 (blood mQTL/eQTL and FinnGen R12 RA GWAS). Scatter plots show consistency between QTL effect sizes and RA GWAS effect sizes for methylation and expression. **(a)** PKD1: blood mQTL (left) and eQTL (right). **(b)** SLC2A4: blood mQTL (left) and eQTL (right).

SMR analyses were performed to assess the association between RA GWAS signals and genetically regulated expression and methylation levels of glycolysis-related genes. Two genes, PKD1 and SLC2A4, showed statistically significant results in both eQTL- and mQTL-based SMR tests. In **Figure 7**, PKD1 (chromosome 16) demonstrated significant colocalization between RA-associated variants and both eQTL (ENSG00000008710) and mQTL (cg07036112) signals (**Figures 7a, b**). Similarly, SLC2A4 (chromosome 17) showed consistent results for eQTL (ENSG00000181856) and mQTL (cg06891043) loci (**Figures 7c, d**). All loci passed the HEIDI test (P > 0.05), and SMR multi-test P-values for each gene were below 0.05. To further evaluate the relationship between QTL effect sizes and RA association signals, SMR EffectPlot analyses were conducted. As shown in **Figure 8**, the effect sizes of top cis-mQTLs and eQTLs for both PKD1 (**Figure 8a**) and SLC2A4 (**Figure 8b**) exhibited linear trends with corresponding GWAS effect sizes. For PKD1, the mQTL probe cg07036112 and the eQTL transcript ENSG00000008710 showed p_SMR_multi values of 0.04058 and 0.01707, respectively. For SLC2A4, cg06891043 and ENSG00000181856 had p_SMR_multi values of 0.00088 and 0.00577, respectively. Top associated SNPs with high linkage disequilibrium (r²) were consistently aligned across datasets. These findings indicated alignment of genetic association signals at both epigenetic and transcriptional levels for PKD1 and SLC2A4 with RA susceptibility loci.

### Whole-blood PKD1 and SLC2A4 mRNA expression in RA versus healthy controls

**Figure 9:**
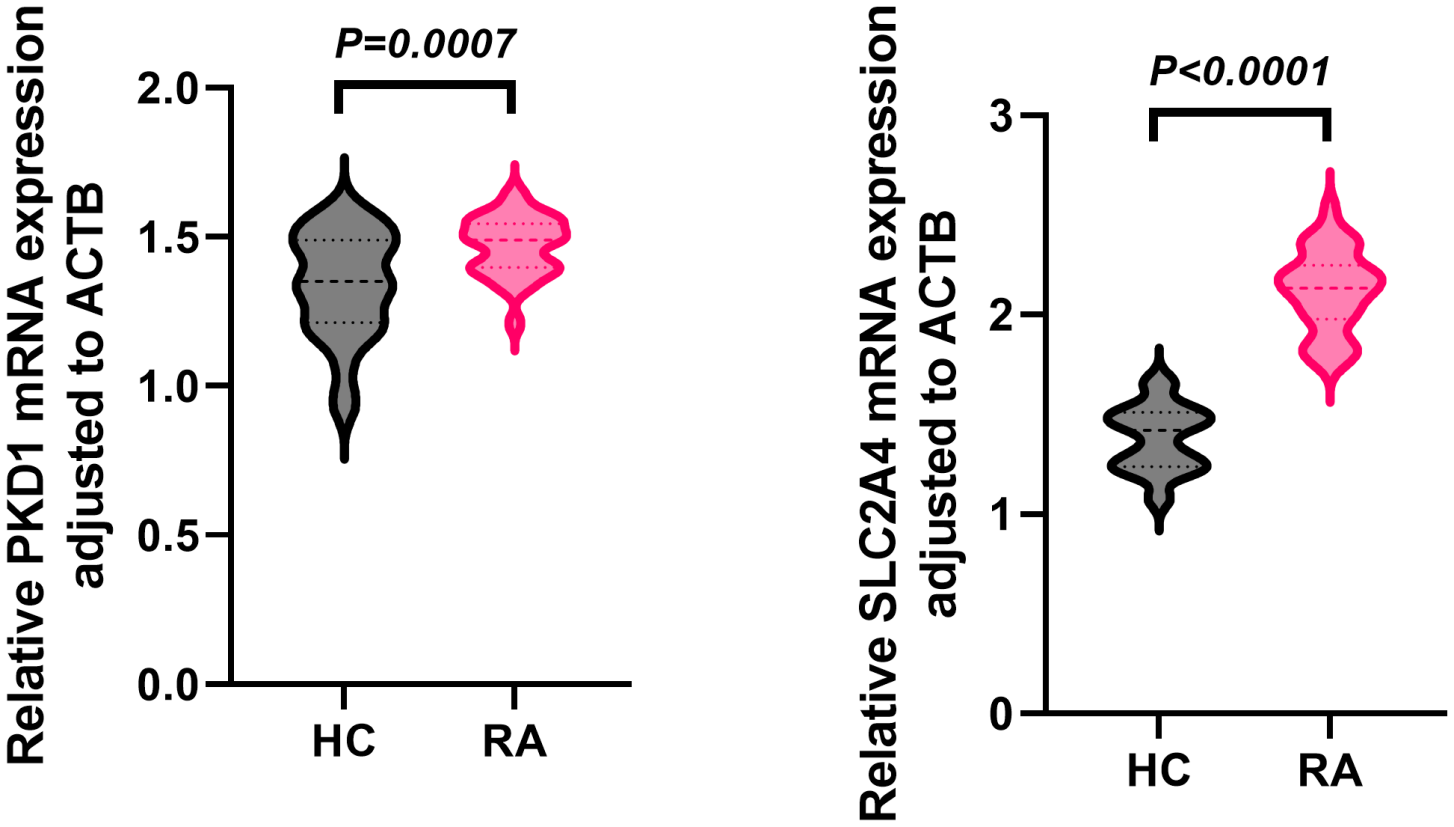
Whole-blood PKD1 and SLC2A4 mRNA expression in RA and healthy controls. Relative expression (2−ΔCt, ACTB-normalized) in RA (n = 30) and healthy controls (n = 30); data are mean ± SD; two-tailed unpaired t-tests.

In the independent whole-blood validation cohort, qPCR analysis demonstrated that both PKD1 and SLC2A4 mRNA levels were significantly elevated in RA patients compared with healthy controls (HC) (**Figure 9**). Relative expression was quantified as 2−ΔCt values normalized to ACTB (β-actin). For PKD1, the mean relative expression was 1.47 ± 0.10 in RA compared with 1.34 ± 0.18 in HC (*P* = 0.0007). For SLC2A4, the mean relative expression was 2.12 ± 0.20 in RA compared with 1.38 ± 0.16 in HC (P < 0.0001).

## Discussion

In this study, we conducted a comprehensive multi-omics MR analysis integrating GWAS with mQTLs, eQTLs, and pQTLs to identify glycolysis-related genes causally associated with RA. Our results revealed significant associations for 129 CpG sites, 28 transcripts, and 9 proteins with RA risk. Among these, 40 CpG sites, 11 transcripts, and 3 proteins showed robust colocalization. Notably, PKD1 and SLC2A4 demonstrated consistent multi-layer associations, supported by replication in independent RA cohorts. Additionally, integrative SMR between mQTLs and eQTLs identified potential epigenetic regulation of glycolysis-related targets, including PYGB, PFKFB2, and ALAS1. These findings support a causal chain from genetic variation through epigenetic regulation and transcriptional alterations to RA susceptibility, strengthening mechanistic links between glycolysis-related regulation and RA.

Notably, several loci contained multiple associated CpG sites within the same gene (e.g., PYGB), and in some cases CpGs within one gene showed opposing directions of association (e.g., JOSD1). Such convergent signals may reflect coordinated, biologically meaningful regional regulation (e.g., promoter/enhancer methylation changes acting in concert to influence transcription). Alternatively, multiple CpG ‘hits’ can arise from correlated methylation structure and linkage disequilibrium at a locus, where nearby probes share the same underlying genetic signal rather than representing independent functional effects. Accordingly, we interpret multi-CpG patterns as supportive of locus involvement but avoid assuming that each CpG has an independent causal role; targeted experimental dissection (e.g., site-specific epigenetic editing) will be needed to resolve CpG-level functionality.

PKD1 emerged as a prominent candidate gene potentially implicated in RA pathogenesis. PKD1 encodes polycystin-1, involved in key inflammatory signaling pathways such as VEGFR2 and NF-κB activation (21, 22). Recent evidence highlighted PKD1’s role in RA synovial inflammation through promoting fibroblast migration and vascular permeability (23). Consistent experimental findings demonstrated that PKD1 knockdown attenuated arthritis severity, further suggesting its pathogenic relevance (24). Interestingly, in cancer studies, PKD1 acts as a tumor suppressor, underscoring its complex context-dependent functions (25). Our SMR analysis revealed a significant causal association between the PKD1 gene and RA risk. Specifically, the methylation site cg07036112 within PKD1 exhibited significant negative regulatory effects in mQTL-eQTL analysis, with methylation levels inversely correlated with PKD1 expression. This negative regulation potentially leads to elevated PKD1 expression, thereby increasing RA susceptibility. Supporting these findings, whole-blood qPCR in an independent cohort demonstrated higher PKD1 mRNA expression in RA patients than in healthy controls, reinforcing its potential as a circulating biomarker.

SLC2A4, encoding the insulin-regulated glucose transporter GLUT4, is classically recognized for its role in metabolic tissues (26). Our MR analysis provided strong evidence linking genetic variants influencing hypomethylation at cg06891043 with increased SLC2A4 expression, consequently increasing RA risk. This finding supports a pathogenic role, where elevated SLC2A4 expression contributes to enhanced glycolytic flux may contribute to increased glucose uptake capacity in RA-relevant immune and stromal pathways, potentially exacerbating inflammation and invasiveness (27, 28). Furthermore, structural and docking studies in oncology contexts indicated that modulation of SLC2A4 might influence glycolytic adaptation and cell survival under inflammatory conditions (29), highlighting its relevance to RA pathology. Therapeutically, interventions targeting GLUT4 expression or its regulatory mechanisms may offer promising strategies to mitigate the metabolic dysfunction underlying RA. Consistent with our genetic and epigenetic results, our whole-blood qPCR validation confirmed increased SLC2A4 mRNA expression in RA patients compared with healthy controls, in line with the direction of effect inferred from the multi-layer MR and QTL analyses.

This whole-blood qPCR validation provides a conceptually aligned translational bridge to our blood-derived mQTL/eQTL evidence by confirming that PKD1 and SLC2A4 transcripts are increased in RA blood. Nevertheless, because this validation is cross-sectional and not genotype-stratified, it does not by itself establish that the observed expression differences are genetically mediated in the same individuals, nor does it resolve cell-type specificity. Future studies integrating paired genotyping with leukocyte subset–resolved transcriptomics and RA-relevant tissues (e.g., synovium) will be valuable to further connect the QTL layer to disease biology.

Other glycolysis-related genes, including PFKFB2, PYGB, and ALAS1, emerged with significant associations. Epigenetic regulation at the PFKFB2 locus suggests modulation of macrophage glycolysis and inflammation resolution mechanisms (30, 31). Similarly, PYGB expression, driven by genetic variation, might sustain cellular energy supply and inflammatory cell survival, paralleling roles previously described in cancer metabolism (32, 33). ALAS1, identified via mQTL-eQTL integration, potentially links mitochondrial heme biosynthesis with autophagic and AMPK signaling pathways essential for cellular survival under inflammatory stress (34). Together, these genes highlight glycolytic pathway complexity in RA, each offering mechanistic insights and therapeutic opportunities.

Functionally, the prioritized genes map onto distinct functional modules of glycolysis-related biology in RA. SLC2A4 (GLUT4) supports glucose uptake, PFKFB2 regulates a key rate-controlling step of glycolytic flux via fructose-2,6-bisphosphate, and PYGB enables glycogen mobilization to supply glycolytic substrates. Together, these findings are consistent with a disease-relevant shift toward increased glucose utilization observed in activated immune cells and pathogenic stromal populations in RA. Although not all highlighted targets are core glycolytic enzymes (e.g., PKD1 may influence metabolic programs via inflammatory signaling), the convergent genetic/QTL evidence supports dysregulated glucose metabolism as an integrated component of RA immunometabolism.

Our study systematically applied multi-omics MR integration, identifying molecular signatures across methylation, expression, and protein levels, surpassing single-layer analyses. Integration of GWAS with mQTL, eQTL, and pQTL datasets prioritized key glycolytic genes including PKD1, SLC2A4, PFKFB2, PYGB, and ALAS1, supported by robust SMR and colocalization analyses (11, 35). This comprehensive approach provided insights into methylation–expression and expression–protein regulatory cascades, exemplifying the methodological strength and robustness of the findings.

Despite the overall consistency of PKD1 and SLC2A4 signals, several other loci identified in FinnGen could not be replicated in the UKB or GCST90129453 RA cohorts. This lack of replication is likely multifactorial. Differences in sample source and collection (e.g. population-based volunteer cohort in UKB vs. health registry-based cohort in FinnGen) and cohort size/power (FinnGen’s >16k RA cases vs. UKB’s ~4.4k) may contribute to diminished signal detection in validation sets. It is also possible that varying phenotype definitions and subtle population genetic differences between cohorts affected the reproducibility of certain associations. We have thus interpreted non-validated loci with caution, recognizing that limited power or heterogeneity across cohorts could explain their absence of replication. This explicit consideration of cross-cohort differences highlights the robustness of the PKD1 and SLC2A4 findings while acknowledging the limitations and context for loci that did not replicate.

Several limitations should be considered. Firstly, our analyses primarily relied on European-derived QTL and GWAS datasets, potentially limiting generalizability to other populations. Secondly, although rigorous methods minimized pleiotropy, residual horizontal pleiotropy in summary-level data cannot be excluded. Thirdly, using blood-derived QTL data may inadequately represent synovial-specific regulatory events. Future tissue-specific and single-cell omics studies are needed. Lastly, our whole-blood qPCR validation was based on a relatively modest sample size, which may limit the precision of the effect estimates and warrants confirmation in larger, independent cohorts. Further validation through diverse populations and functional experiments remains necessary.

In conclusion, this integrative multi-omics MR analysis identifies PKD1, SLC2A4, and additional glycolysis-related genes as putative causal regulators of rheumatoid arthritis. By linking genetic variation to epigenetic and transcriptional changes associated with RA risk, these findings clarify mechanisms of metabolic reprogramming and nominate candidates for biomarker development and metabolism-targeted therapy, including potential repurposing of modulators of PKD1 or GLUT4. Looking forward, longitudinal cohorts, multimodal omics, and artificial intelligence/machine learning (AI/ML)–enabled analytics may refine causal inference, improve patient stratification, and accelerate precision medicine in RA.

## Data Availability

The raw data supporting the conclusions of this article will be made available by the authors, without undue reservation.
