# Correction: Integrative multi-omics analyses identify PKD1 and SLC2A4 as genetically supported glycolysis-related candidate genes for rheumatoid arthritis

**DOI:** 10.3389/fimmu.2026.1793746

**Published:** 2026-01-29

**Authors:** Xinyu A, Pengfei Xin, Lin Zheng, Bo Xu, Jianye Wang, Songtao Sun, Jun Xie, Chenxin Gao, Peijun Pan, Guowei Qiu, Lang Jin, Jun Shen, Xirui Xu, Yiwei Cheng, Shaoqiang Pei, Lei Ran, Yanqin Bian, Lianbo Xiao

**Affiliations:** 1 Shanghai University of Traditional Chinese Medicine, Shanghai, China; 2 The Research Institute for Joint Diseases, Shanghai Academy of Traditional Chinese Medicine, Shanghai, China; 3 Jiangxi University of Traditional Chinese Medicine Affiliated Hospital, Nanchang, China; 4 Shanghai Guanghua Hospital of Integrative Medicine, Shanghai, China

**Keywords:** DNA methylation, gene expression, glycolysis, Mendelian randomization, multi-omics, PKD1, rheumatoid arthritis, SLC2A4

An incorrect number was provided for the National Natural Science Foundation of China. The correct number is “(82474302)”.

The funder the Changning National Master of Traditional Chinese Medicine Studio was erroneously omitted. The Funding statement has been corrected to read:

“The author(s) declared that financial support was received for this work and/or its publication. This work was funded by the National Natural Science Foundation of China (82474302), Changning District Science and Technology Commission (CNKW2024Y19), the Training Program for High-caliber Talents of Clinical Research at Affiliated Hospital of SHUTCM (2023LCRC24), Shanghai Municipal Health Commission (20244Y0010), Scientific Research Project of Changning District Science and Technology Commission (CNKW2022Y22) and Shanghai Science and Technology Commission Support Project (23Y11921900), and was also supported by the Changning National Master of Traditional Chinese Medicine Studio”.

The original version of this article has been updated.

