# Supplementary figures and images for: Integrative multi-omics analyses identify PKD1 and SLC2A4 as genetically supported glycolysis-related candidate genes for rheumatoid arthritis
Source: Front Immunol. 2026 Jan 22;16:1691663. doi: 10.3389/fimmu.2025.1691663 (PMC12895683; doi:10.3389/fimmu.2025.1691663)

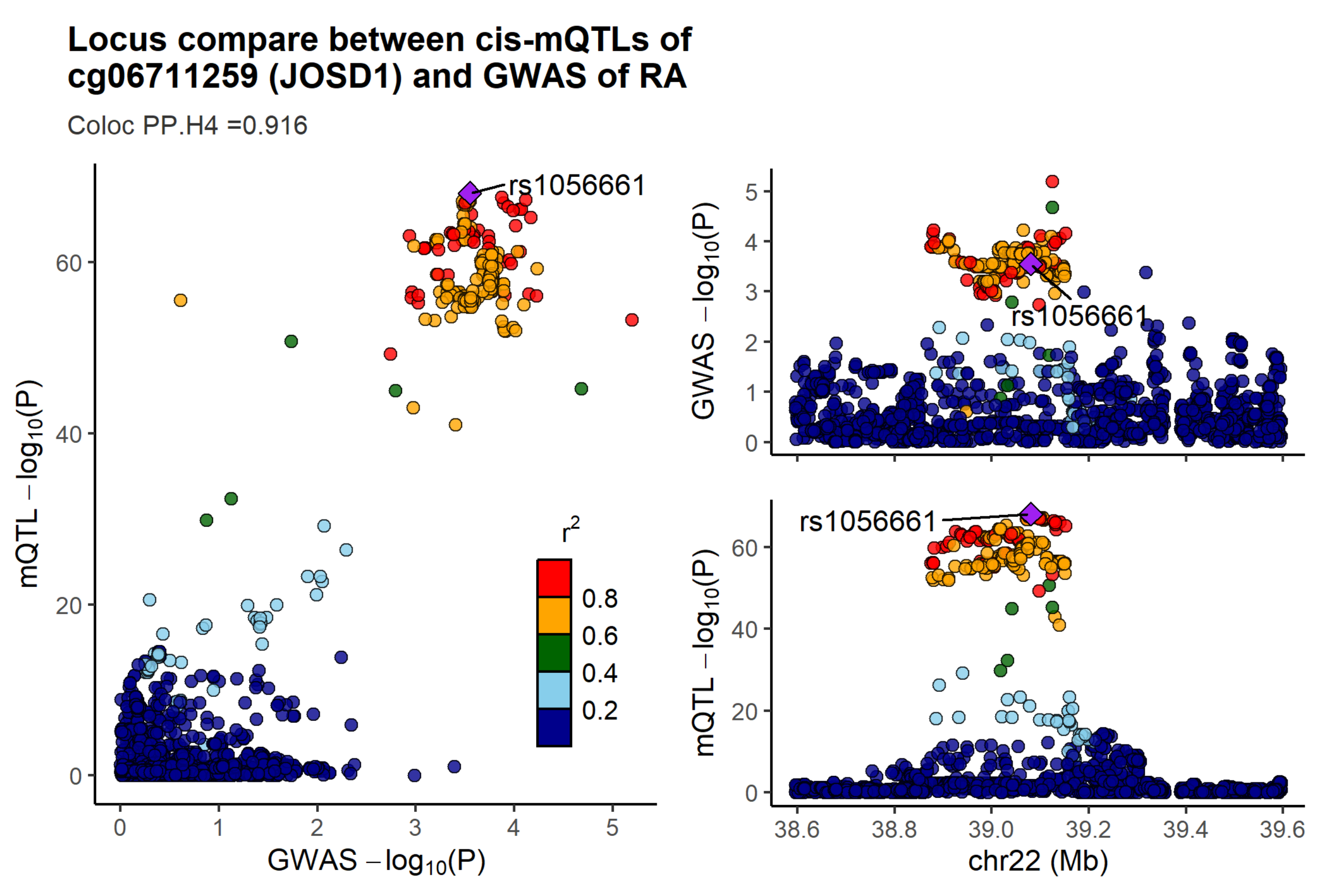

Supplement: Supplementary file 1 [file Image1.tif]

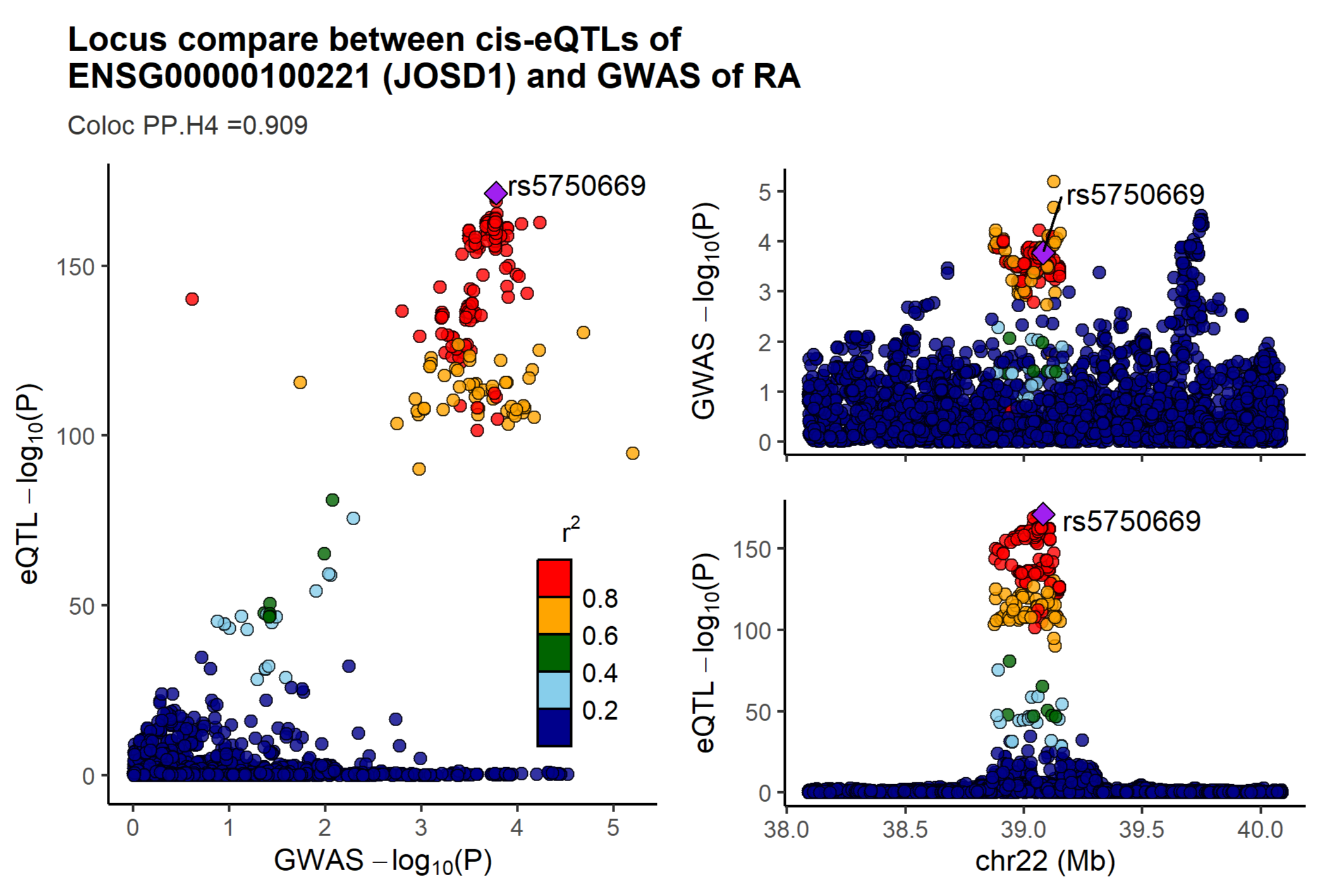

Supplement: Supplementary file 2 [file Image2.tif]

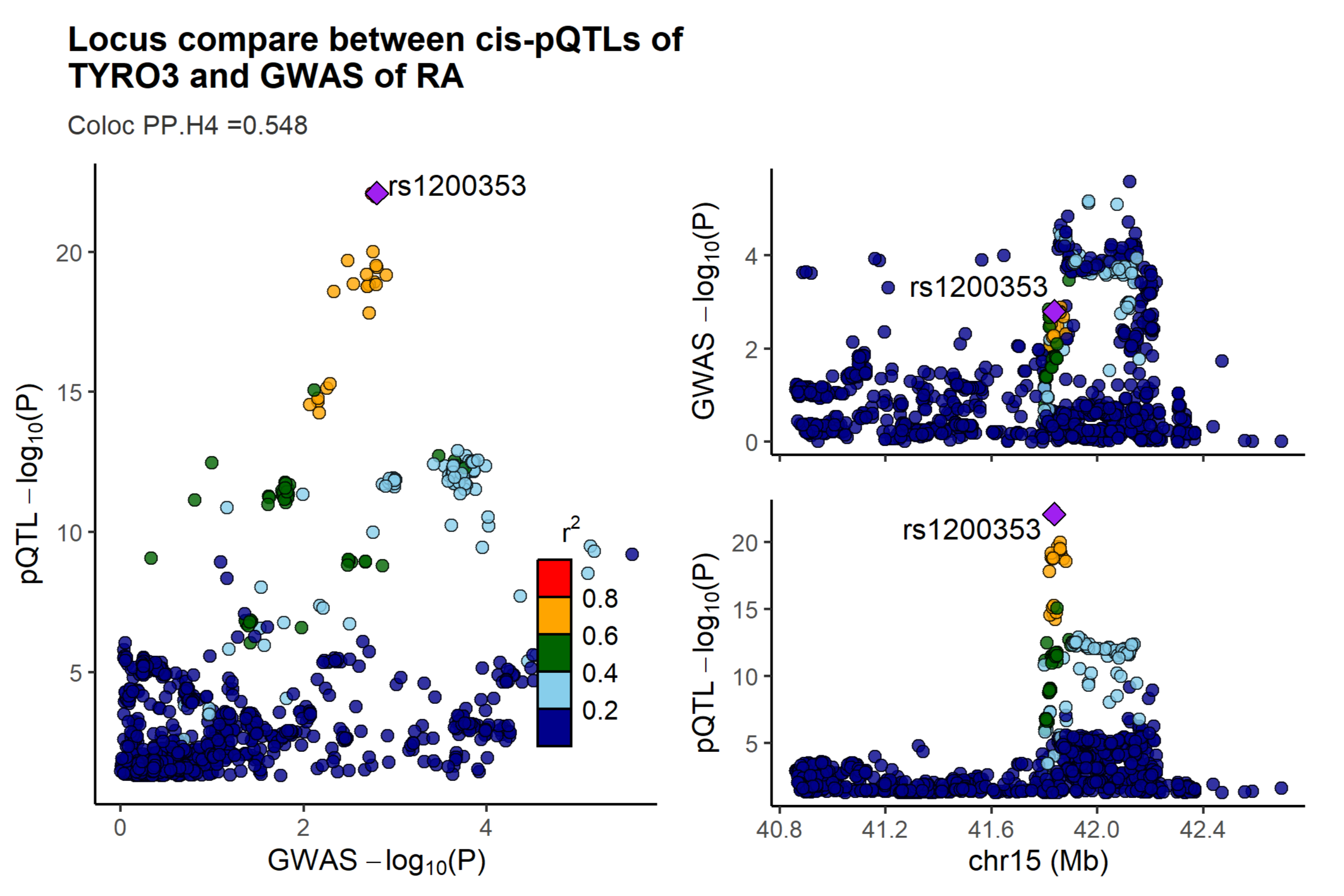

Supplement: Supplementary file 3 [file Image3.tif]
